# A control theoretical approach to gene regulation reveals quantitative constraints for dynamic homeostasis in stochastic gene expression^☆^

**DOI:** 10.1016/j.bbadis.2026.168219

**Published:** 2026-03-13

**Authors:** Guilherme Giovanini, Cyro von Zuben de Valega Negrão, Ammar Alsinai, Marsha Rich Rosner, Gábor Balázsi, Alexandre Ferreira Ramos

**Affiliations:** a Departamento de Radiologia e Oncologia, Instituto do Câncer do Estado de São Paulo (ICESP), Hospital das Clínicas da Faculdade de Medicina da Universidade de São Paulo (HCFMUSP), São Paulo 01246-000, SP, Brazil; b Brazilian Biosciences National Laboratory (LNBio), Brazilian Center for Research in Energy & Materials (CNPEM), Campinas 13083-970, São Paulo, Brazil; c Department of Computer Science, College of Engineering and Information Technology, Onaizah Colleges, Unaizah 56447, Qassim, Saudi Arabia; d Ben May Department for Cancer Research, The University of Chicago, Chicago 60637, IL, United States; e The Louis and Beatrice Laufer Center for Physical and Quantitative Biology, Stony Brook University, Stony Brook 11794, NY, United States; f Department of Biomedical Engineering, Stony Brook University, Stony Brook Cancer Center, Stony Brook 11794, NY, United States; g Comprehensive Center for Precision Oncology, Instituto do Câncer do Estado de São Paulo (ICESP), Hospital das Clínicas da Faculdade de Medicina da Universidade de São Paulo (HCFMUSP), São Paulo 01246-000, SP, Brazil; h Escola de Artes, Ciências e Humanidades, Universidade de São Paulo, São Paulo 03828-000, SP, Brazil

**Keywords:** Two-state stochastic gene, Regulator gene model, Feedback control, Bursty gene expression

## Abstract

Cell phenotype dynamic homeostasis contrasts with the inherent randomness of intracellular reactions. Although feedback control of regulator genes (RG) is a key strategy for limiting the range of downstream gene expression, understanding the quantitative constraints and corresponding mechanisms enabling such a dynamic stability under noise remains elusive. Here we model RG expression as a stochastic process and downstream genes as sensors whose responses conditionally induce RG activity. We show that at homeostatic regime: *i.* the trajectories of the RG expression levels can be adjusted towards specific ranges using both the exact solutions of the stochastic model and the exact stochastic simulation algorithm (SSA); *ii.* there exists a sampling rate which optimizes the feedback control of the RG activity, and non-optimal controls resulting in alternative homeostatic dynamics; *iii.* the feedback control of RG activity leads to updates whose intensities and time intervals are non-linearly related; *iv.* the ON state probability of an RG promoter has dynamics confined within a narrow domain. Our results help to understand the quantitative constraints underpinning dynamic homeostasis despite randomness, the mechanisms underlying alternative, non-optimal, homeostatic regimes, and may be useful for theoretically prototyping therapies aiming at gene network modulation.

## 1. Introduction

Understanding how homeostasis is reached despite the inherent randomness of the molecular processes underpinning cell dynamics remains a major challenge of the post-genomic era. The presence of reactants in low copy numbers inside the cell causes random fluctuations in the number of reaction products [1] while homeostasis requires a more constrained dynamical adaptation of the intracellular machinery in response to environmental stimuli [2]. In the dynamical systems parlance, the latter corresponds to a trajectory surrounding an attractor despite parameter value variability. Random fluctuations, however, have been characterized as a source of phenotypic changes [3–5] which may drive the dynamics of the system towards alternative attractors corresponding to diseased states [6].

Hence, maintaining gene expression levels around an optimal average is crucial for the stability of regulatory networks [7]. For instance, subtle fluctuations in the expression of regulators of epithelial–mesenchymal transition (EMT) can determine whether cells remain epithelial or change their phenotype towards invasive states [8]. A mechanism to prevent random transitions to alternative attractors is negative feedback control as it provides phenotypic homeostasis [9], and a strategy for regulating noise of biochemical processes and their cellular consequences [7,10–14]. Feedback control is often implemented in a gene network whose dynamics is modulated by the expression levels of a regulator gene (RG) and its multiple targets [9,15]. Since a plethora of systemic diseases, such as cancers, are associated with disruption of a standard homeostatic profile of living systems towards alternative ones [3,6,16], understanding the mechanisms governing the dynamical stability of cells is key for the design of more effective therapeutic strategies [5,17,18].

For that, a powerful approach is to design therapies as control problems [19] formulated to take the inherent stochasticity of the intracellular environment into consideration [20]. That strategy aids in characterizing the mechanisms of homeostasis in response to stochastic processes. Hence, we propose coupling a feedback control and a two-state stochastic model [21] for a RG as the latter has been used to model bursts of gene expression in mouse and human fibroblasts [22,23]. Our framework sheds light on the quantitative constraints and mechanisms governing the homeostasis of a RG. Furthermore, it provides a prototype for the development of a control theoretic-based formulation of gene therapy for cancer and other diseases.

**Fig. 1.**
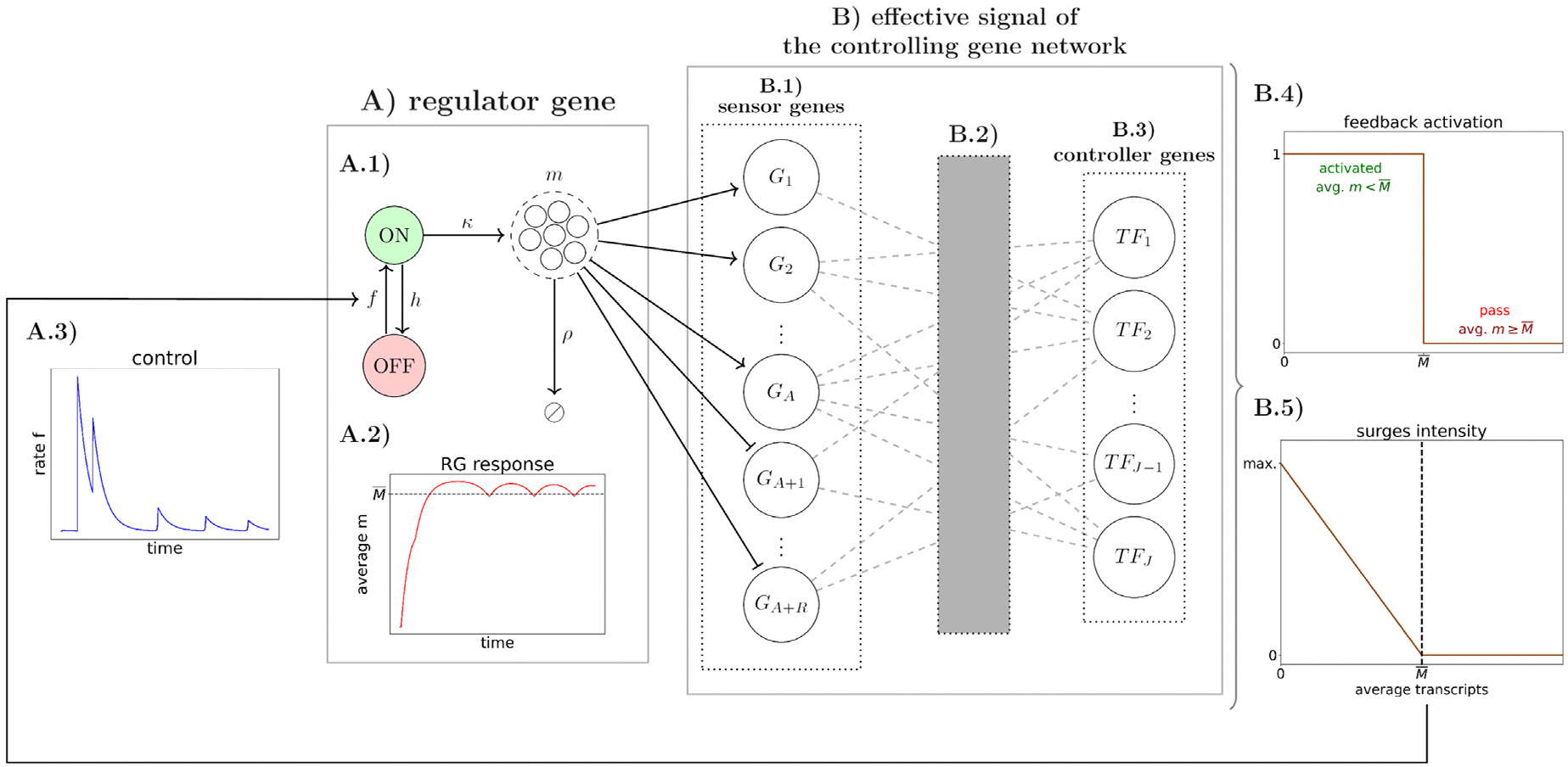
A system composed by a regulator gene (RG) and its controlling gene network underpins cellular phenotype homeostasis. We denote the number of products at instant t synthesized from the RG by m. The promoter of the RG switches between states ON and OFF with effective rates f and h. The average duration of the OFF state (~1/f) is modulated by the controlling gene network which emits an effective signal constituted by amounts of its genetically encoded transcription factors (TF) ($TF_{1},\ldots ,TF_{J}$). Note that m is the input which modulates the signal emitted by the controlling gene network. RG products-sensitive genes sense m. Their response, measured as gene expression levels, is communicated through the network, changing the TF expression profile. The gray intermediate layer of the network represents the plethora of genes that can participate in the signal propagation. The cellular phenotype is a readout of the collective state of our system. The aimed phenotype state is proxied at a target level of RG expression, $\bar{M}$, to activate and adjust the feedback surge intensity.

**The phenomenology of our formulation**, depicted in Fig. 1, is useful for understanding our framing of the problem. The cellular phenotype is set in terms of the RG expression level m in comparison to an arbitrary reference value $\bar{M}$. The biological assumptions underpinning that choice will be discussed later. Fig. 1A indicates the RG modeled as a two-state system (see Fig. 1A.1). Fig. 1A.2 shows the average expression of the RG in response to the control acting on the switching rate from OFF to ON state denoted by M. The value of the latter is modulated by a feedback effect that is turned on whenever the sensing detects $m<\bar{M}$. The control dynamics is shown in Fig. 1A.3. The choice of f as a target for the control was inspired by experiments with *E. coli* [24,25] and D*. melanogaster* [26] showing that the duration of the OFF state is the gene-parameter being modulated. In bacteria, f can be changed by inducers such as IPTG [27] while ectopic misexpression is typically used for changing ON state durations in *Drosophila* embryos [28]. Then, the OFF to ON switching rate can be associated to a single molecule or to the effective action of a group of molecules governing promoter accessibility. Fig. 1B indicates the target genes with which the RG RNAs interact directly or indirectly. The set of target genes comprise a gene network with a somewhat cumbersome architecture in which first and last layers, respectively, contain the sensor (Fig. 1B.1) and controller (Fig. 1B.3) genes. The gene network topology is explicitly established for specific systems [9,29–31]. Here we consider an abstract gene network, and the gray box (Fig. 1B.2) highlights arbitrary connections. Fig. 1B.4 shows that the feedback mechanism is activated when $m\;<\;\bar{M}$ while the magnitude of the surges aiming to modulate f are displayed in Fig. 1B.5. The products of the genes being affected by the RG RNAs are typically synthesized in bursts [22,23,32], as is the sensing enabled by them. To mimic this effect, we assume that m is sampled with a constant average period.

Using the model depicted in Fig. 1 we show: *i.* that the trajectories of the amounts of RNAs from the RG can be adjusted towards specific values using either the exact solutions for average RNA transcripts, ⟨m⟩(t), obtained from the two-state model [33] or the algorithm for exact stochastic simulations of chemical reactions [34]; *ii.* the existence of a sampling rate which identifies stability transitions of RG homeostatic dynamics and optimizes the feedback surges controlling the activity of the RG; *iii.* the feedback surges generated by net effect have a nonlinear relationship between the intensities and time intervals and; *iv.* the dynamics of the probability for the promoter of the RG being ON is confined within a narrow domain when homeostasis is reached. The presented model provides insights into how homeostasis emerges despite randomness, and indicates that rebalancing the time scales of the sensor system enables redirecting the homeostatic regime of a RG.

### Biological justification of the arbitrary modeling choices.

As a model system of an RG, we chose the metastasis suppressor gene Raf Kinase Inhibitory Protein (RKIP) [35,36]. RKIP is a kinase modulator that directly binds to Raf-1 kinase and inhibits the MAP kinase network in breast cancer [37]. RKIP indirectly participates in signaling pathways that modulate transcription factors and ultimately influence cell phenotype [38,39]. The Raf–MEK–ERK pathway regulates the phosphorylation of a myriad of transcription factors by ERK, and dephosphorylated RKIP directly binds to Raf-1, thereby preventing Raf-1 activation and attenuating ERK signaling. The NF-*κ*B pathway is also reported to be negatively regulated by RKIP, which interferes with IKK activation and reduces NF-*κ*B-dependent transcription [40]. Other signaling pathways modulated by RKIP include G-proteins, the Keap1/NRF2 redox-sensing switch, STAT3 and GSK-3 [41]. Therefore, maintaining pathways regulators such as RKIP at near-optimal levels may aid in preserving downstream network stability, either preventing undesired phenotypic switching, or re-establishing near-normal cellular functions. Indeed, the formulation of a low-dose multi-drug treatment targeting MAP kinases that mimics normal RKIP expression enabled metastatic suppression [37,42]. In that formulation the control, *i.e.* treatment, was implicitly assumed to be activated when $m\;<\;\bar{M}$ because of the action of RKIP as a metastasis suppressor.

Regulation of RKIP transcript levels may occur through histone-mediated epigenetic mechanisms. Methylation of histones at RKIP promoter sites is correlated with reduced gene expression in several cancer types [43,44], whereas treatment with histone deacetylase inhibitors can restore RKIP levels [45,46]. In contrast, the transcription factors Snail and BACH1 repress RKIP transcription by recruiting histone methyltransferases [47,48]. Both factors are central drivers of the epithelial–mesenchymal transition (EMT): Snail directly suppresses E-cadherin transcription, promoting loss of cell–cell adhesion [49], while BACH1, a basic leucine-zipper protein broadly expressed in mammalian tissues, induces motility-associated genes that facilitate metastasis in breast cancer [50]. Expression of Snail and BACH1 is itself negatively regulated by RKIP, creating reciprocal inhibitory loops. Furthermore, BACH1 and RKIP form a bistable regulatory circuit that operates as a molecular switch governing metastatic phenotype acquisition in tumor cell populations [48]. To represent regulation of RKIP by TFs Snail and BACH1, we selected the rate f to be affected by the feedback control, similar to our choice in a previous study [20].

The remainder of this manuscript is organized as follows: in Section 2, we describe the theoretical formulation for the coupling between the stochastic binary model for a RG, and the transcripts-dependent feedback control of RG expression by the gene network effect. Our results about the control of homeostatic RG expression and emergent behavior resulting from parameter values of the control are presented in Section 3. Results are discussed in Section 4 while Section 5 presents the limitations of our approach and our conclusions.

## 2. Methods

### Phenomenological coupling of feedback control to a two-state stochastic model for regulation of gene transcription.

We model a RG using the exactly solvable two-state stochastic model [21,51], which is widely used as a basic building block to understand noise in gene expression [22,33,52–57]. The qualitative features of the two-state model for stochastic gene expression is depicted within the regulator gene–box in Fig. 1. It has two random variables (s,m), where s∈{ON,OFF} denotes the state of the promoter, and m∈{0,1,2,…} is the number of RNAs. The promoter randomly switches from state OFF to ON (and vice-versa) with a rate f (and h). The synthesis and degradation rates are respectively denoted by κ and ρ, with synthesis only happening when the promoter is ON. We also introduce a modification: the number of transcripts is monitored with a sampling rate ν, and if the number of transcripts is lower than a pre-set aimed value, one or more rates governing the state of the system should be changed to drive expression back to the aimed level $\bar{M}$. Here we choose to only increment the value of, as that choice provides sufficient intricacy for clearly demonstrating our theoretical formulation.

The aforementioned effective chemical reactions are summarized from Eqs. (1)–(5). Let us denote by: ℛ, the promoter of the gene, 𝒫 the balance of products from the sensing genes producing a net effect which may (or may not) induce an increase on OFF to ON rate (f) of the RG; and ΔRNA, the aimed change on the number of RNAs to restore its expression level towards its aimed value $\bar{M}$. The latter transition aims to rebalance 𝒫 by an amount Δ𝒫. That sets a gene network functioning as a feedback system incrementing f as indicated by the balance on the number of products synthesized from the target genes. Functional transcripts of RG are represented by RNA and degradation of, or loss of functionality by, RNAs is indicated by ⊘:

(1) $$ℛ+𝒫\overset{f(𝒫)}{⇀}ℛ𝒫,$$

(2) $$ℛ𝒫\overset{h}{⇀}ℛ+𝒫,$$

(3) $$ℛ𝒫\overset{\kappa }{⇀}ℛ𝒫+RNA,$$

(4) $$RNA\overset{\rho }{⇀}⊘,$$

(5) $$𝒫\overset{v}{⇀}𝒫+\Delta 𝒫\left(\Delta RNA\right).$$

Eqs. (1) – (5) respectively indicate promoter switching from OFF to ON, and ON to OFF, synthesis and degradation of RNAs, and the sampling of RNAs number. The rate f≡f(𝒫) indicates that a differential configuration on the number of products from the target genes will affect the OFF to ON switching rate (Fig. 1).

**The time-dependent solutions of the two-state model with constant parameter values** can be used for simulating the dynamics of our system after performing a proper piecewise decomposition of the time-domain. The functions governing the evolution of the moments of the probability distributions governing (s,m), for constant parameter values, have been presented elsewhere [33]. Let us set the parameters $\epsilon ,\;A_{s}$ and N, respectively, as the ratio of the gene switching rate between ON and OFF states and the degradation rate of the gene products, the steady state probability for the promoter to be ON, and the steady state average number of products for a promoter fully ON:

(6) $$\epsilon =\frac{f\;+\;h}{\rho },\;A_{s}=\frac{f}{f\;+\;h},\;N=\frac{\kappa }{\rho }.$$

The parameter N sets the maximum capacity of transcription of the RG. It is an effective representation of the whole transcriptional process, from the binding of the RNAPolII to the promoter site to the synthesis of a mature RNA. $A_{s}$ is the steady state fraction of time during which the promoter stays in the ON state. That reflects the interaction of the regulatory binding sites and the TFs.

For parameter values of Eq. (6) being constant, one may write the equations governing the dynamics of the probability for the promoter being ON, A(t), and the average number of products, ⟨m⟩(t), as

(7) $$A(t)=A_{s}+\left(A_{0}-A_{s}\right)e^{-\epsilon \rho t},$$

(8) $$\langle m\rangle (t)=\langle m\rangle_{s}+Y\;e^{-\epsilon \rho t}+V\;e^{-\rho t},$$

where $A_{0}=A(0)$, and $\left\langle m\rangle_{0}=\langle m\rangle (0\right)$ are initial conditions, $\langle m\rangle_{s}=NA_{s}$ is the steady state average number of gene products, $Y=N\frac{A_{0}\;-\;A_{s}}{1\;-\;\epsilon }$, and $V=\langle m\rangle_{0}-\langle m\rangle_{s}-Y$. The gene transcription constitutive regime at maximal rate corresponds to the RG promoter being always ON and ⟨m⟩=N.

#### Partitioning the time domain to enable the use of the exact solutions.

Because of the feedback, the parameter f becomes time-dependent (f→f(t)), and hence, the solutions at Eqs. (7) and (8) lose their usefulness. To recover that, we recently proposed a piecewise decomposition of the time-domain of a simulation of the system [20]. The length of each piece is set to ensure an arbitrarily small difference between a constant $\bar{f}$ and f(t) within the sub-interval. Then, the exact solutions A(t) and ⟨m⟩(t), at Eqs. (7) and (8) can be used within each time interval setting the state of the solution at the end of a time-piece as the initial condition of the next one. Here on f(t) is eventually denoted by f, with the temporal dependence being inferred from context.

### An approach for investigating homeostatic gene expression by feedback-based modulation of the OFF to ON switching rate.

The feedback is based on sampling instants at which m is sensed by the controlling gene network. The assumption of discontinuity presumes that the components sensing the RG products are synthesized in bursts [22,23,58,59], i.e., their availability for interacting with the RG products is intermittent. We use the dynamics of the moments to set our mathematical model and, hence, we use an average rate of surge of sensing elements (the sampling rate v) instead of random sensing events. In that picture, the sampling instants $T_{n}$ can be set as $T_{n}=T_{0}+\frac{n}{v}$, where $T_{0}$ is the instant of the first sampling, and n ranges from 0 to the last sampling.

To illustrate our methodology, we consider a gene whose expression level in a normally functioning cell must be high. The parameter values are set to drive a steady state regime having low expression levels when the feedback system is not operational. That condition corresponds to a RG being strongly repressed within a cell operating in an abnormal regime. Hence, as an initial condition we assume the RG being in a low expression level steady state regime set in terms of a small OFF to ON switching rate $f_{s}$. The feedback surges will operate to increase f(t) and, consequently, the number of transcripts (see Eq. (1)).

We assume that the effect of a feedback surge on f(t) decays exponentially with rate λ, and that f(t) returns to $f_{s}$ [20]. Hence, f is considered an effective rate of reaction resulting from either the amount of TFs available for controlling the RG or the duration of their binding to the DNA. That is the simplest experimentally grounded modeling of the temporal dependence of f since exponential decay is a first-order model that fits well both the degradation of multiple protein classes [60] or TF dissociation and turnover from DNA [61]. More elaborated temporal dependencies of f, such as biexponential or power law distributions of residence times could be proposed under the assumption of more complex TF binding models [62].

In our simulations, we have an initial time interval from 0 to $T_{0}$ during which the system is in a steady state regimen characterized by $\langle m\rangle /\bar{M}=0.1$. At $t=T_{0}$ we have the first sampling of ⟨m⟩. After a fixed time interval T=1/v there will be a sensing event to verify the conditional increase of f. This change is assumed to be sufficiently fast to be approximated as instantaneous such that the update in f, when needed at $T_{n}$, is:

(9) $$f_{n}\equiv f\left(T_{n}\right)=f_{n}^{*}+\Delta f_{n}$$

where $\Delta f_{n}$ is a positive number proportional to $\langle m\rangle (t)-\bar{M}$, n=0,1,…, and $f_{n}^{*}$ is obtained from the evolution of f(t) during the time interval between two samplings:

(10) $$f\left(t\right)=f_{s}+\left(f_{n}-f_{s}\right)e^{-\lambda \left(t-T_{n}\right)},\;for\;T_{n}\leq t<T_{n+1},$$

more precisely, $f_{0}^{*}=f_{s}$ for $t<T_{0}$ and $f_{n}^{*}={lim}_{t\to T_{n}^{-}}f(t)$ for $T_{n-1}\leq t<T_{n}$. The instantaneous increment assumption is because of the phenomenology of the controlling gene network which produces the TFs regulating the RG. We consider that the TFs are produced in bursts, which presumes that synthesis is much faster than the remaining effective phenomena. That is the simplest possible model for the TFs regulating stimuli-responsive genes. Including additional effects such as TF cytoplasmic activation, state switching, and nuclear translocation would lead to an alternative formulation of $f_{n}$ [63].

### A phenomenological proposal for updating f.

As a first order approximation for proposing a feedback control, we consider ⟨m⟩(t) to be in a hypothetical steady state value at instants $T_{n-1}$ and $T_{n}$. Hence, this implies on assuming the average number of RNAs being approximated by the hypothetical steady state values $f=f_{n-1}$ and $f=f_{n}$. Accordingly with Eq. (8) the averages $M_{n-1}$ and $M_{n}$ are

(11) $$M_{n-1}=\frac{\kappa }{\rho }\frac{f_{n-1}}{f_{n-1}\;+\;h},$$

(12) $$M_{n}=\frac{\kappa }{\rho }\frac{f_{n-1}\;+\;\Delta f_{n}}{f_{n-1}\;+\;h\;+\;\Delta f_{n}}=\bar{M}.$$

The feedback increment $\Delta f_{n}$ is obtained from the difference $\Delta M_{n}=M_{n}-M_{n-1}=\bar{M}-M_{n-1}$ which is a positive quantity as $M_{n}$ is always set to be equals to $\bar{M}$. Then, the OFF to ON switching rate in Eq. (9) is changed by:

(13) $$\Delta f_{n}=\frac{h\;+\;f_{n-1}}{N\;-\;\bar{M}}\Delta M_{n},$$

which is always positive because we assume that $N>\bar{M}$. It implies considering that the transcriptional apparatus – encompassing promoter binding by RNAPolII, RNA elongation, processing, and turnover – is not significantly changed because of disease. Thus, the cell remains potentially capable of maintaining $\sim \bar{M}$ transcripts by modulating the duration of the ON and OFF states of the promoter. Here, f is modulated to reduce the OFF state duration proportionally to $\Delta M_{n}>0$ while f decays exponentially (without modulation) when the amount of transcripts is larger than $\bar{M}$. A biologically feasible pathologic condition is to have $N<\bar{M}$ [64,65] because of promoter methylation. Then, one would control the RG synthesis rate, κ=Nρ [20], by means of treatment based on a demethylation agent [66].

**The stochastic simulation algorithm (SSA),** or Gillespie algorithm [67], is a rigorous approach for performing exact simulations of the evolution of systems of chemical reactions. The dynamics produced by the effective chemical reactions shown in Eqs. (1)–(5) were obtained using the SSA. Eq. (5) indicates the reaction that monitors the number of gene products to produce the increments in f by means of feedback. That is the stochastic analog of the procedure adopted to insert a feedback in the averages as shown in Eq. (9). In the SSA scheme, we also assume an exponential decay and random instantaneous increments of f, and v as the propensity of occurrence of the reaction (5).

### Dynamics of the ON state probability and average number of transcripts as a control system.

The two-state model for gene regulation with time-dependent f is governed by the following system of coupled ODEs [20]:

(14) $$\frac{dA(t)}{dt}=-\left(f\left(t\right)+h\right)A\left(t\right)+f\left(t\right),$$

(15) $$\frac{d\langle m\rangle (t)}{dt}=-\rho \left\langle m\right\rangle \left(t\right)+\kappa A\left(t\right).$$

Formulating as a linear control system we have, $\dot{x}(t)=𝒜(t)x(t)+ℬ(t)u(t)$, where the state vector x(t) and input u(t) are respectively:

(16) $$x\left(t\right)=\left[\begin{array}{c} A\left(t\right)-A^{*}\\ \langle m\rangle (t)-{\left\langle m\right\rangle }^{*} \end{array}\right],\;u\left(t\right)=\left[\begin{array}{c} f\left(t\right)(1-A^{*})-hA^{*},\\ 0 \end{array}\right].$$

$A^{*}$ and ${\left\langle m\right\rangle }^{*}$ are arbitrary constants set as the ON probability and the corresponding average at some given instant. The matrices 𝒜(t) and ℬ(t) denote the homogeneous and non-homogeneous components of the system, and using Eqs. (14) and (15) we obtain:

(17) $$𝒜\left(t\right)=\left[\begin{array}{cc} -\left(f\left(t\right)+h\right) & 0\\ \kappa & -\rho \end{array}\right],\;ℬ\left(t\right)=\left[\begin{array}{ll} 1 & 0\\ 0 & 0 \end{array}\right],$$

where ℬ enables the control.

The state of the system at t, x(t), is obtained by the action of a transition matrix ${\tilde{\Phi }}_{𝒜}\left(t,t_{0}\right)$ related to the matrix 𝒜 on $x\left(t_{0}\right)$. The solution x(t), which exists and is unique, can be computed by means of the celebrated Dyson series (also known as Peano–Baker series) [68,69]. The closed form of that solution for time-dependent rates is beyond the scope of the current study. Because we are investigating the phenomenology underlying the homeostasis of cellular phenotype using the simplest possible theory for regulation of stochastic gene expression, a numerical analysis of the solutions suffices.

Notice, though, that we do have the closed forms of the solution for constant kinetic parameters which will be useful in our numerical computations. Let us partition the time domain in L+1 subintervals, namely $\left[t_{0},t\right]=\left[t_{0},t_{1}\right)\cup \left[t_{1},t_{2}\right)\cup \ldots \left[t_{L},t_{L+1}\right]$. During each $\delta_{l}=t_{l+1}-t_{l}$ we may assume that all kinetic parameters are constants within an arbitrarily defined precision, so that $f_{l}\equiv f\left(t_{l}\right)$, is computed using Eq. (10). Because we presume an exponential decay of the effect of the control onto a given kinetic rate, the length of the subintervals vary (see Ref. [20] for a description). During each subinterval $\left[t_{l},t_{l+1}\right)$, the transition matrix is:

(18) $$\Phi_{𝒜}\left(t_{l+1},t_{l}\right)=\left[\begin{array}{cc} e^{-\left(f_{l}+h\right)\delta_{l}} & 0\\ \frac{\kappa \left(e^{-\left(f_{l}+h\right)\delta_{l}}-e^{-\rho \delta_{l}}\right)}{\rho -\left(f_{l}+h\right)} & e^{-\rho \delta_{l}} \end{array}\right].$$

The composition property of transition matrix, $\Phi_{𝒜}\left(t_{l+1},t_{0}\right)=\Phi_{𝒜}\left(t_{l+1},t_{l}\right)\Phi_{𝒜}\left(t_{l},t_{0}\right)$, enables the piecewise approach to perform small parameter variation within 𝒜.

The controllability of the system is assessed by means of the eigenvalues of 𝒜. During each $\delta_{l}$ the matrix has two eigenvalues $-\left(f_{l}+h\right)$ and -ρ with

(19) $$\left[\begin{array}{c} 1\\ \frac{\kappa }{\rho -\left(f_{l}+h\right)} \end{array}\right],\left[\begin{array}{l} 0\\ 1 \end{array}\right]\;$$

being their respective eigenvectors. Since $f_{l}$, h, ρ are positive real, all eigenvalues are negative real, ensuring that the system is exponentially stable (see Theorem 6.10 in [69]). The controllability matrix $\left[\begin{array}{ll} ℬ & 𝒜ℬ \end{array}\right]$ has full rank and, hence, the system is controllable (Theorem 9.5 [69]). Considering these theoretical results for the time-invariant system, we will act onto f parameter to investigate the stability and controllability of x(t).

## 3. Results

We simulate the trajectories of the number of transcripts starting from the initial condition $\langle m\rangle_{s}=10$. The feedback surges were set to cause a 10-fold increase in the number of gene products, i.e. from 10 to 100. The sampling frequencies, v, underpin the surge of the feedback span from 1 × 10^−2^ to $2\times 10^{2}\rho$. For simplicity we set $\rho =1{TU}^{-1}$, where TU denotes the time unit corresponding to the half-life of the transcripts which is used to set the time scale of our system. The values of the kinetic rates $\left(f_{s},h,\kappa \right)$ are (0.9, 9.1, 110) in units of ${TU}^{-1}$. These parameters characterize a quasi-Poissonian probability distribution governing the RNA number in the steady-state regime [20,70]. Using Eq. (6), the distributions can also be characterized by another auxiliary set of phenomenologically interpretable parameters: $\left(\epsilon ,A_{s},N\right)=(10,0.09,110)$. Before the beginning of the feedback surges, we consider that the system is in a steady-state regime. The first feedback surge occurs at instant 1 TU, and we follow the dynamics of the system until $t=10^{3}\;TU$. The intensity of the effect of the first increment is 90 for all trajectories because of the aimed 10-fold increase in ⟨m⟩. To investigate how the decaying rates of the effects of the feedback surges affect the control, we use the following values of λ: (0.01, 0.1, 0.5, 1, 2). The time steps of the dynamics are computed using a piecewise approach applied to the exponential decay function f(t), as described in [20], where the absolute error of each subinterval and the stopping criterion, $f(t)-f_{s}$, are both 1 × 10^−8^.

### 3.1. Sampling rate enables regulation of homeostatic RNA levels

**Fig. 2.**
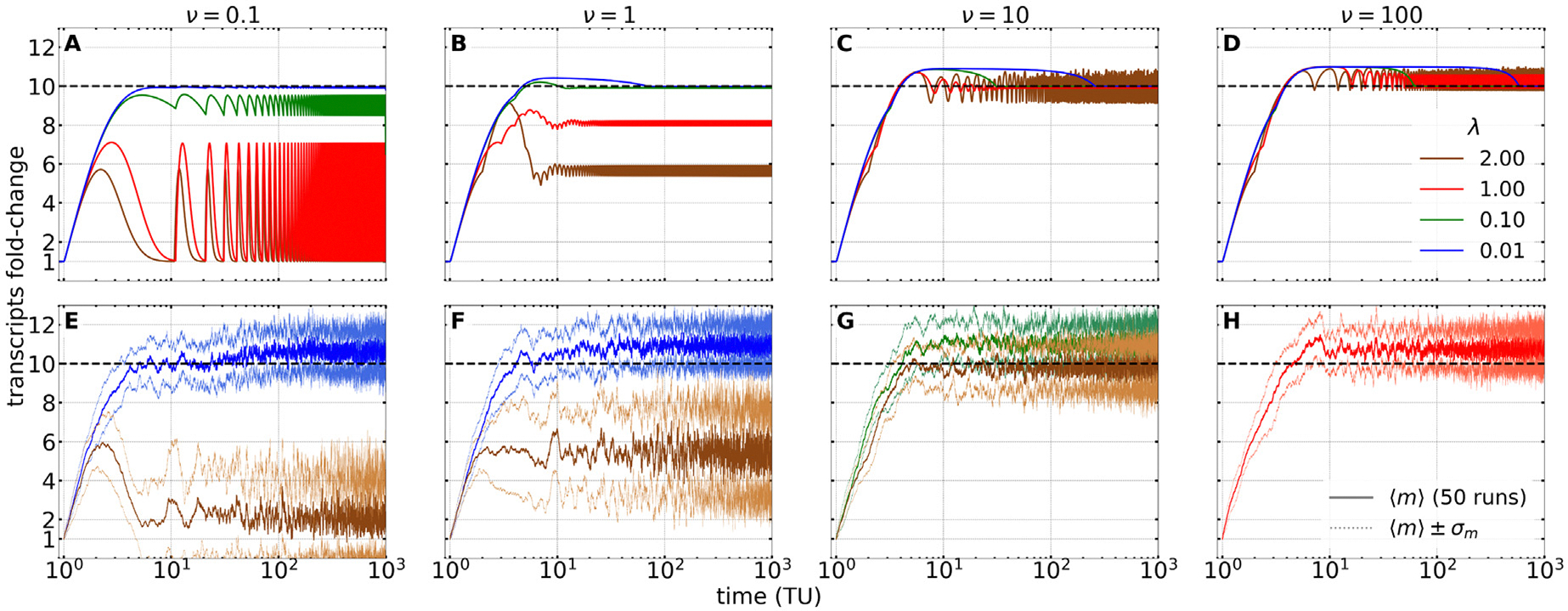
Sampling rate regulates RNA levels at homeostasis. From left to right, the columns present trajectories with increasing sampling rate v. All curves follow the color key within **(D)** for four different decaying rates of increment effect λ. **(A-D)** depict analytical trajectories of the fold-change in the average RNA levels ⟨m⟩. **(E-H)** show average and standard deviation of RNA numbers computed by 50 runs using the Gillespie algorithm. A dashed line at 10 indicates the fold-change corresponding to $\bar{M}$. The scales of the rates and time are relative to the RNA degradation rate ρ.

Fig. 2 shows the dynamics of the fold-change of the average numbers of transcripts computed using either Eq. (8) (top row) and fifty trajectories obtained by the SSA simulation of the reaction scheme of Eqs. (1)–(5) (bottom row). We used four sampling rate values (see columns) and four decaying rates of the feedback effect (color key in graph **(D)**). For a sampling at time $t=T_{n}$, the amount of RNAs ⟨m⟩(t) is compared to the aimed value $\bar{M}$. For $\langle m\rangle (t)<\bar{M}$ the feedback surges and f(t) is incremented by $\Delta f_{n}\propto \Delta M_{n}$ (Eq. (13)) to induce an increase in the number of transcripts. Fig. S1 in Supporting Information (SI) displays 5 trajectories of m(t) obtained with SSA to illustrate the variability of transcription dynamics.

The increase of ν stabilizes ⟨m⟩(t) around $\bar{M}$ whether we use the analytically obtained, or SSA dynamics, typically with reduced variability. In graph **(A)**, the average number of transcripts reaches homeostasis for λ=0.01. For v≥1, graphs **(B-D)**, the trajectories for λ<1 exhibit dynamics that overshoot and then dampen towards the $\bar{M}$. For λ≥1, the homeostatic regime shows an oscillatory-like behavior. As v increases, the oscillations surround $\bar{M}$, with more heterogeneous amplitudes and a larger band. Lower sampling rates, v=0.1, do not allow the ⟨m⟩(t) dynamics to stabilize when λ≥0.1. Note, however, the existence of trajectories exhibiting a dynamic homeostasis around intermediary values of ⟨m⟩ which are regulated by the relation between ν and λ. This behavior is also observed in the trajectories obtained using Gillespie algorithm.

Comparing the analytical curves with SSA ones, it is noticeable that for v=0.1, **(A)** shows fast increases on the amount of transcripts, while in **(E)**, the curve with larger λ shows less controllable behavior (brown curve). When v=1, the peak that appears around t=3TU in **(B)** for λ=2 (brown curve) does not occur in graph **(F)**, because only a few trajectories obtained by SSA tend to rise at this instant (SI Appendix, Fig. S1 – Graph **(B)**). For v≥10, the amplitudes of the bumps in the number of transcripts become more similar in the analytical and SSA curves. Because the SSA trajectories behave similarly to those obtained from the analytical solutions, we use the analytical solutions to analyze the properties of the feedback control proposed by us. Note, however, that our theoretical analysis will show results that can be extrapolated for the realization of the stochastic process as simulated by the SSA and, consequently, on the analysis of experimental data.

### 3.2. Mapping timescales of model

This subsection presents two analyses of the homeostasis of the expression of a RG. The effect of tuning the sampling and feedback surge decay rates to the homeostasis is analyzed in terms of (ν,λ) and in terms of ν/λ. Put together, the results show transitions of homeostatic dynamics among stable regions, and revealed optimal parameter tuning for the effects of feedback surge.

#### Identifying stability transitions of homeostatic dynamics dependent on feedback surge decay and sampling rates.

**Fig. 3.**
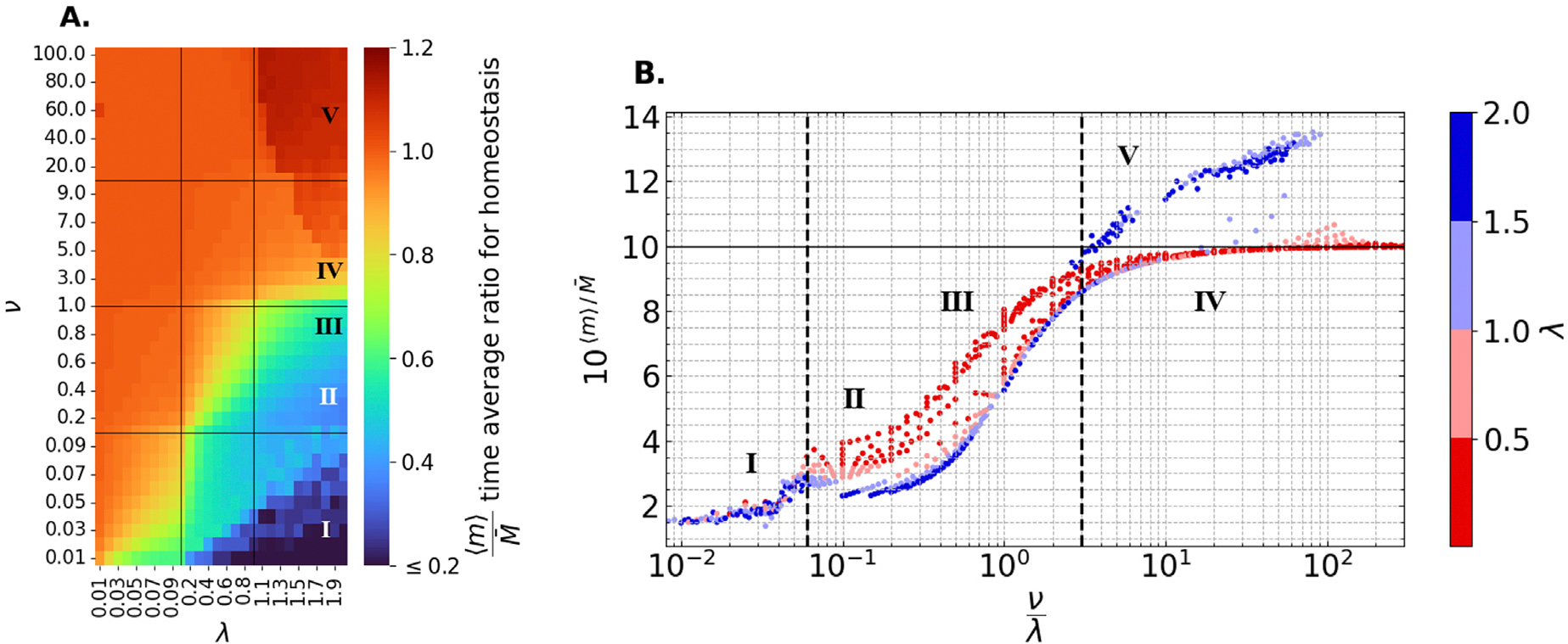
Dependence map of homeostatic dynamics on feedback surge decay and sampling rates. The heatmap displayed in **Graph (A.)** presents the dependence of $\langle m\rangle_{H}/\bar{M}$ to (λ,v). The ratio values are coded by color bar. Solid black lines indicate a change of scale. Five dynamical regimes are shown: non-homeostatic **(I)**, transitional non-homeostatic to homeostatic with $\langle m\rangle_{H}\ll \bar{M}$ (**II**); homeostatic (**III**) with $\langle m\rangle_{H}\sim 0.8\;\bar{M}$, (**IV**) with $\langle m\rangle_{H}\sim \bar{M}$ and (**V**) with $\left.\langle m\rangle_{H}\right\rangle \bar{M}$. This coarse-grained setting of the regimes are strongly related to the value of ν. The scatter plot displayed in **Graph (B.)** shows the relationship between $\langle m\rangle_{H}/\bar{M}$ and v/λ. The *y*-axis shows $\langle m\rangle_{H}/\bar{M}$ as the exponent of 10 for detailed visualization. The horizontal solid line indicates $\langle m\rangle_{H}=\bar{M}$ and the dashed ones the two transition points at v/λ~0.06 and ~3.

Fig. 3 presents the dynamical regime of transcripts levels regulation determined by the temporal average of the mean number of RNAs during homeostasis, $\langle m\rangle_{H}$, depending on λ and ν. Graph A. shows a heatmap for the ratio of $\langle m\rangle_{H}$ to the aimed value $\bar{M}$. The analysis of homeostasis is performed for t≥900TU. Graph B. displays a scatter plot for $\langle m\rangle_{H}/\bar{M}$ versus v/λ. The conditions λ≤1 and λ>1 are indicated, respectively, by reddish and bluish dots. The distinguishable dynamical regimes were identified with roman numerals.

The heatmap of $\langle m\rangle_{H}/\bar{M}$ as function of the rates λ and v in Graph **A.** show non-linear transitions between dynamical regimes. For instance, λ>1 generates five types of trajectories, where homeostasis at $\langle m\rangle_{H}\sim 0.8\;\bar{M}$, (**III**) is confined within the neighborhood of v~1. For 0.1<λ≤1, the region of the non-homeostatic regime (**I**) is shrunk towards the lowest values of v. Homeostasis with $\langle m\rangle_{H}\ll \bar{M}$ regime (**II**) is maintained for v<0.1. Regimes (**III**) and (**IV** – homeostatic with $\langle m\rangle_{H}\sim \bar{M}$) span respectively within ranges: 0.1≤v≤1 and v>1, while the overshot homeostatic regime (**V**) does not occur. For λ≤0.1, (**IV**) is predominant for almost all ν values except for very low ones.

Graph **B.** shows the λ values separating the five regimens in terms of v/λ. For v<0.06λ, $\langle m\rangle_{H}/\bar{M}$ is in regime (**I**), and the dynamics for high and low λ are similar. $\langle m\rangle_{H}/\bar{M}$ for v≤0.05λ and v<3λ is in (**II**) and (**III**), with low λ values leading to $\langle m\rangle_{H}\sim \bar{M}$. For v≤3λ, $\langle m\rangle_{H}\sim \bar{M}$ splits into two regimes: (**IV**) for low λ and (**V**) for high ones.

#### Sampling rate reveals an optimum for the average feedback surge effects.

**Fig. 4.**
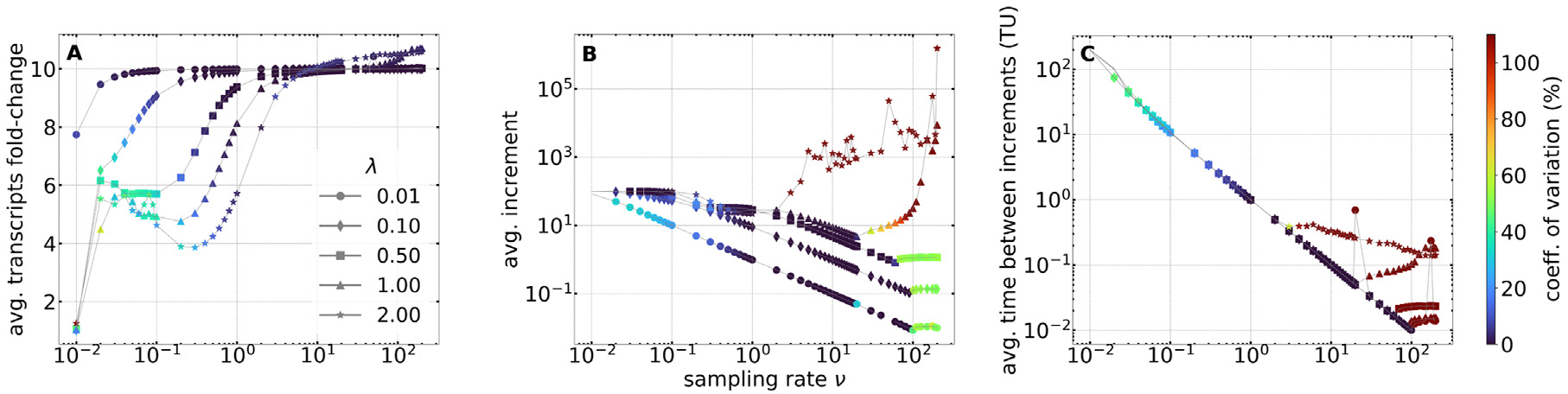
Optimal sampling rates minimizing average feedback surges intensities. The graphs depict the dependency of three variables on the sampling rate: the average fold-change in RNA levels **(A)**, the average intensity **(B)**, and the time interval **(C)** of the increment effect for dynamics at the homeostatic regime. Marker colors indicate the coefficient of variation (CV) in percentage for each respective *y*-axis variable. Rate and time scales are relative to the RNA degradation rate ρ. All x-axes and the **(B–C)** y-axes are in logarithmic scale.

Fig. 4 has the sampling rate v at horizontal axes while the vertical axes of graphs (**A**), **(B)**, and **(C)**, respectively present, at the homeostatic regime, the average fold change of ⟨m⟩, the intensities of increments of feedback surges and time intervals between these increments. The homeostatic regime is defined for t≥900TU. This interval ensures that for larger v, all ⟨m⟩ trajectories fluctuate within a defined band approaching the aimed level. The color code on the right indicates coefficient of variation (CV) values of the variables of the corresponding vertical axis. Each symbol style indicates a different value of λ as shown in graph **(A)**.

Graph **(A)** shows that ⟨m⟩ reaches $\bar{M}$ for larger v. It is noteworthy that ⟨m⟩ exceeds $\bar{M}$ for v>50 when λ=1, and for v≥10 when λ=2. For v<10, ⟨m⟩ decreases and its CV increases for all λ. Local minima of ⟨m⟩ fold-change are observed for ν between 0.04 and 0.3. The minimum at the lowest ν occurs for λ=0.5 with ⟨m⟩ approximately 5.5 -fold; and the one at the highest ν occurs for λ=2 with ⟨m⟩ around 4-fold. For λ≤0.1, ⟨m⟩ decreases monotonically as ν decreases.

Graph **(B)** shows the decrease in average increment as v increases up to a λ-dependent threshold. From the lowest to the highest λ, the minimum average increment occurs for v=(100,90,60,20,2). The CV of increment effects increases for v higher than that minimum one, and the average increment becomes higher and unstable for λ≥1. In this case, increments surpass those of lower v.

In graph **(C)**, as v increases, the average intervals between increments form a descending straight line until they reach a threshold. From the smallest to the second-largest λ, the respective v that minimizes the average time intervals are (100, 100, 60, 20). Note that for λ=2, no minimum occurs, and in this case, the time intervals decrease slowly for v>3. Similar to (**B**), if v is larger than the threshold, the CV of the increment interval increases.

### 3.3. A nonlinear relationship between time intervals and intensities of feedback surges

**Fig. 5.**
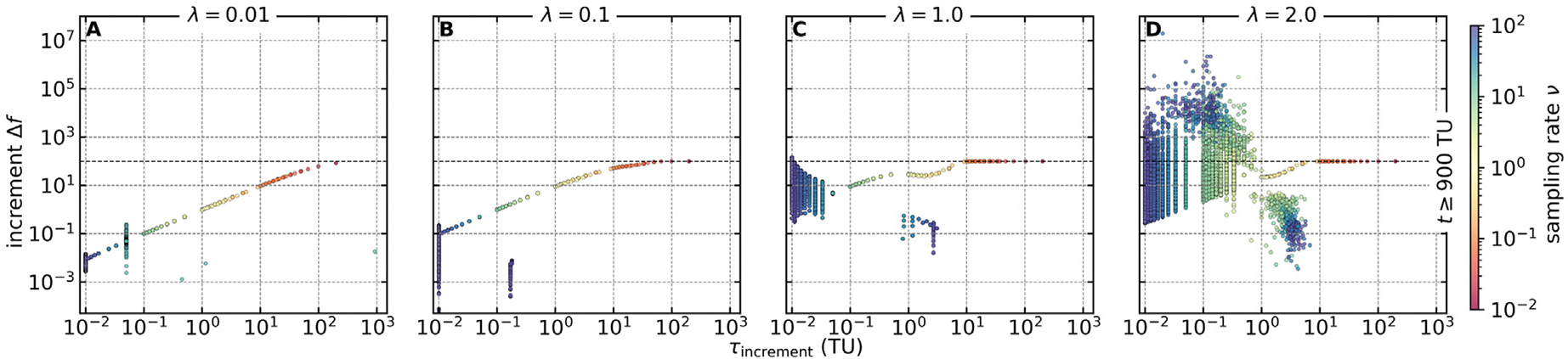
A nonlinear relationship between time intervals and intensities of feedback surges in homeostasis. **(A–D)** show scatter plots of feedback surges space for the homeostatic regime, t≥900TU, namely, in the *y*-axis the increment effects and in the *x*-axis the interval between these increments. The dots’ color indicates the sampling rates v corresponding to the color code located to the right of the figure. Decaying rates λ for each graph are displayed at the column top. All axes are in logarithmic scale, and the rates and time are relative to ρ.

The scatter plots in Fig. 5 depict the space of feedback surges. Graphs **(A-D)** display overlapping data points representing dynamics at homeostatic regime for t≥900TU. They were computed for a wide range of sampling rates v, equally spaced in logarithm scale from 10^−2^ to 10^2^. In SI Appendix, Fig. S2, it is shown the complete time series of the time intervals and intensities of feedback surges for v>1.

For decaying rate λ=0.01, graph **(A)**, the feedback surges agenda forms a straight line exhibiting a wide range of linear dependence between increment Δf and $\tau_{\text{increment}}$. Note that agendas with lower sampling rates, v<1 (reddish dots), exhibit a more regular pattern. However, for v≥1 (greenish to bluish dots), the increments become smaller and some variability begins to appear. For example, when v≥ 10, some agendas which increments vary up to 3 orders of magnitude. As λ increases, graphs **(B)** to **(D)**, the linear relation between Δf and $\tau_{\text{increment}}$ becomes a non-monotonic curve. For v<1 (yellowish to reddish dots) the increment intensities are fixed at 90. For higher v, the heterogeneity in the agenda increases. Homeostasis is established by increments with: i. small increments and intermediate $\tau_{\text{increment}}$, *ii*. large increments and low $\tau_{\text{increment}}$, and *iii.* highly variable increments with low $\tau_{\text{increment}}$.

### 3.4. The dynamics of the probability for the promoter to be ON is confined within a small domain during homeostasis

**Fig. 6.**
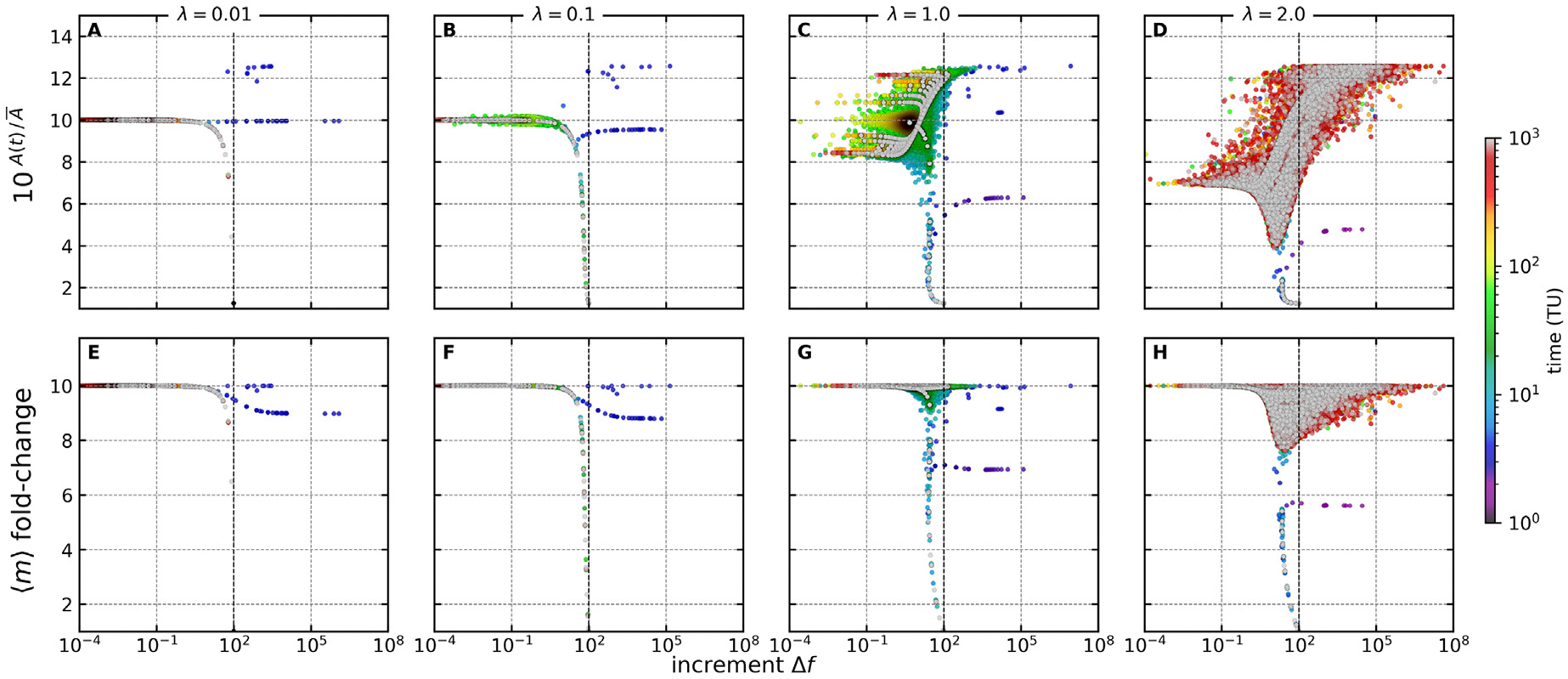
Probability for the promoter to be ON and respective average RNA fold-change during homeostasis. **(A–D)** and **(E–H)**, depict, respectively, the time course of the dependence between increment intensities and the ON state probability, A(t) or the respective fold change in the average RNA levels, ⟨m⟩(t), for a comprehensive range of sampling rate ν. The marker colors represent time in TU (see the color code on the right). The gray points indicate the homeostatic regime. The horizontal axes are displayed in logarithmic scale, while the vertical ones denote both the normalized ON-state probability and the fold-change in ⟨m⟩. The normalized probabilities of the ON-state are better visualized through rescaling to powers of 10.

Fig. 6 depicts the relation of the feedback surges effect on f to: the gene promoter ON state probability, graphs (**A-D**); the average fold change in RNA levels, graphs (**E-H**). We consider a wide range of sampling rates v. The feedback surges are computed using Eq. (13) while the ON probability A(t) and ⟨m⟩(t) levels are determined by Eqs. (7) and (8), respectively. Here, the aimed ON probability $\bar{A}$ for a 10-fold increase in RNA levels is 0.9. It is worth noting that $10^{A(t)/\bar{A}}=10$ implies $A(t)=\bar{A}$. The first increment always has an intensity of 90 such that $10^{A/\bar{A}}=1.26$.

When λ≤0.1, in graphs (**A-B**) and (**E-F**), the early increments (bluish dots) exhibit intermediate and high intensities, with $A\geq \bar{A}$ and ⟨m⟩ fold-change varying between 8 and 10 -fold. During the transient (greenish and reddish) and homeostasis (gray) regimes, low increment intensities occur keeping $\langle m\rangle \;\sim \;\bar{M}$ and $A(t)\;\sim \;\bar{A}$. Intermediate increments occur when A(t) and ⟨m⟩ are far from the aimed value. For λ=0.1, increments in the transient regime fluctuate around $\bar{A}$. In homeostasis, A(t) and ⟨m⟩ become more heterogeneous as λ increases, accessing more values below the aimed one — compare graphs **(A, E)** and **(B,F)**.

For λ=1 (graphs **(C)** and **(G)**), the increment intensities shift to the intermediate region between 10^−1^ and 10^2^. Early increments maintain the intensity, however they may occur less effectively, with A(t)≪1 and ⟨m⟩ fold-change around 7. Two behaviors of feedback surges are revealed in the time course: *i*. the increment intensities concentrate around 10 with A(t) and ⟨m⟩ close to aimed value, *ii*. the intensities and ON probability ranging between two states maintain RNA levels around $\bar{M}$, namely, low intensity around 10^−1^ and $10^{A(t)/\bar{A}}\approx 8(A(t)=0.82)$, and the state with intermediate intensity around 10^2^ and $10^{A(t)/\bar{A}}\approx 12(A(t)=0.98)$.

For the changing of λ value from 1 to 2, graphs **(D)** and **(H)**, the behavior of feedback surges observed in aforementioned case *i*. is changed with the attractiveness to a central point during homeostasis being transformed towards case *ii.* However, in this scenario, ⟨m⟩ fold-change is less effective and a new minimum appears at $10^{A(t)/\bar{A}}\approx 4$ with an increment intensity 10^1^ (see **(D)**). Note that in the homeostatic regime, both the range of increment intensity and the distance between the probabilities of two states (case ii.) increase, RNAs levels may be lower, ~8-fold, and the feedback surges become more heterogeneous: lower increments vary between 10^−2^ to 10^1^ while higher increments reach up to ~10^6^.

## 4. Discussion

The feedback-based control model for modulating the expression of a RG sheds light on the conditions underlying homeostasis as a property emerging from the coupling of multiple stochastic processes having distinctive time scales [71,72]. Our prototypic model enables governing the dynamics of a system that encompasses four effective processes whose respective timescales are set by the : *i.* RNA degradation rate ρ; *ii.* gene switching frequency ε; *iii.* decaying rate of the feedback effect λ; *iv.* sampling rate ν. Timescales *i.* and *ii.* drive the two-state stochastic model for gene regulation, *iii.* depends on the mechanisms of the feedback control, and *iv.* can be determined in terms of the sensors which are being affected by the products of the RG [9].

Our approach provides a strategy and a framework to investigate cellular phenotype reprogramming, a key goal of advanced cancer therapies [37,42]. Indeed, the existence of alternative homeostatic regimens of expression of a RG are shown (Fig. 2B and F). That illustrates a condition for the emergence of diseases characterized by the rebalance of gene expression levels of RGs: they may result from changes of the gene network timescales that drive the cells to assume a damped homeostatic functioning.

Introducing the feedback mechanism prevents building an intervention agenda as recently reported by us [20]. However, the fine tuning of the control still requires analyzing the parameter space of the model for properly modulating the expression of the RG towards a specific aim. We show that setting the control by the average number of RNAs replaces Gillespie’s SSA on the search for homeostatic regimens (Fig. 2). Hence, seeking for proper reaction rates becomes computationally cheaper and enables the use of more complex optimization techniques such as simulated annealing [73,74].

The effectiveness of the feedback-based control on providing homeostatic dynamics has a strong dependence on the sampling rate. Indeed, larger values of the sampling rate cause late feedback surges to happen at small deviations from the aimed average number of RNAs (Fig. 2D). At this limit, tiny changes in f(t) are sufficient for correcting the trajectories for all values of λ (Fig. 2D). The earlier feedback surges cause larger increments (Fig. S2) and responses which overshoot $\bar{M}$ (Fig. 2D). The variability of the feedback surges parameters is increased — see CV in Fig. 4B and C, and the increment size time series in Fig. S2. The increments in f(t) and the interval between feedback surges approach a minimum as v increases (Fig. 4B and C). Both the increment intensity and interval between surges have minima for smaller values of λ. But when λ=2 the minimum exists only for the increment. As the sampling rate is reduced towards the degradation rate of the RNAs, the role of λ, the decaying rate of the effect of the feedback control on f(t), becomes more prominent. E.g., for v≤1, the response in RNA levels to feedback surges is not effective for λ≥0.5 (Figs. 2A–B, 4A). The sampling rates v≥1 provide homeostatic expression levels at the aimed value. When λ>0.1 feedback surges show two behaviors: short spaced in time along with highly variable increment; and widely spaced in time along with fixed size increments (Fig. 5C and D). In these cases, the ON state gene promoter probability A(t) is v-dependent, as v>10, the behavior of A(t) changed from a single stable state value around $\bar{A}$ to an oscillatory one (Fig. 6C and D). As λ increases the dynamics of the control become more complex because of the augmented heterogeneity of ⟨m⟩(t) (Fig. 6G and H).

## 5. Conclusions and limitations

Bursty gene expression has been widely recognized as a source of randomness inside cells [22,23,75–77]. Reconciling that stochasticity with the robustness of cellular phenotype determination remains as a major challenge of the post genomic era. The approach presented in this manuscript shows that a feedback control established using the model for the average number of products synthesized from a two-state gene enables its expression to be homeostatic. That is also verified using Gillespie’s algorithm. Our result provides a strategy for aiding in settling the apparent paradox between randomness and robustness in biological systems.

The approach presented here opens up an avenue for further investigating both the mechanisms of homeostasis and homeostatic-retrieval treatment design. The theory for regulation of gene expression used here lacks important effects such as RNA elongation, protein synthesis, explicit gene-gene interaction, or cell division. The latter, for example, might require the target gene expression levels to be adaptable as cell division causes the amounts of RNAs to present a somewhat oscillatory behavior [32]. Additionally, experimental results on changes of the rates of synthesis [66], ON to OFF switching [78], and (or) degradation of RNAs [79–81] suggests designing the control to target all rates of the model [20].

Our framework can be applied to investigate RKIP expression levels recovery in metastatic cells by the action of gene-parameter readjustment-drugs. For instance, [48] reports that the RKIP - let 7 - BACH1 loop has two stable cellular phenotypes, anti- and pro-metastatic, which are respectively marked by high and low concentrations of RKIP. The homeostasis of the anti-metastatic regimen is maintained under small alterations of the parameters of the loop. A transition towards the metastatic state is induced only by large variations of the parameters of the loop. That is a strong candidate for testing our modeling approach as one might design a therapy for reestablishing RKIP levels through transcriptional regulation [66]. An idealized scenario may also be considered. Assume one may stably introduce an extra RKIP therapeutic gene into every cell of a tumor to prevent metastasis. Because cells tend to naturally resist transgene expression, some of them would start silencing the exogenous RKIP rendering the therapy increasingly ineffective. Suppose it is possible to monitor the downstream effects of RKIP in the whole tumor by bulk average of some kinase levels or the expression of some genes. That indicates RKIP levels lowering and demand intervention, *e.g.* by means of a small molecule inducer such as doxycycline. In this context, doxycycline-inducible gene systems inserted into genomic safe harbor sites, which tend to avoid silencing, provide strategic technology for achieving controlled RKIP expression [82]. Our model aids in determining the frequency of the monitoring to ensure that RKIP remains at therapeutically necessary levels.

Additionally, because our framework provides tools for investigating the mechanisms of homeostasis and cellular phenotype engineering, it may also be tested using alternative systems. For instance, Ronin (Thap11) underpins the formation of hubs of promoters which lead to the collective regulation of the expression of housekeeping genes [83]. Hence, Ronin might be modeled as a RG to aid in understanding the dynamics of metabolism and growth of embryonic stem cells [84]; cardiogenesis [85]; and aging-related illnesses [86]. Feedback gene network architectures have been experimentally characterized in a variety of systems. Direct negative auto-regulation modulates about 40% [87,88] of the known transcription factors encoded by *E. coli* while long feedback loops characterize the cholesterol catabolism sensing system in Mycobacterium [29,31]. Direct negative feedback loops provide stability to gene networks [7,10], linearize and speed up response to input signals [11,13], and represent a strategy for noise reduction [14]. Engineering long feedback leverages from natural systems to build, for instance, integrated circuits of sensors of multiple mutated RAS expressing a specific protein prone to be used as a proxy for cancer cells [89] or optogenetic feedback control to sustain gene expression levels in bacterial systems [90].

## Supplementary Material

1

## Data Availability

Data will be made available on request.

## Appendix A. Supplementary data

Supplementary material related to this article can be found online at https://doi.org/10.1016/j.bbadis.2026.168219.

## References

[1] Delbrück M, Statistical fluctuations in autocatalytic reactions, J. Chem. Phys. 8 (1) (1940) 120–124, 10.1063/1.1750549.

[2] Balk B, Goodrich DW, The molecular determinants of phenotypic plasticity in homeostasis and neoplasia, Cancer Heterog. Plast (2024) 10.47248/chp2401020010. PMC12455573 40994652

[3] Guinn MT, Wan Y, Levovitz S, Yang D, Rosner MR, Balázsi G, Observation and control of gene expression noise: Barrier crossing analogies between drug resistance and metastasis, Front. Genet 11 (2020) 10.3389/fgene.2020.586726. PMC7662081 33193723

[4] Desai RV, Chen X, Martin B, Chaturvedi S, Hwang DW, Li W, Yu C, Ding S, Thomson M, Singer RH, Coleman RA, Hansen MMK, Weinberger LS, A dna repair pathway can regulate transcriptional noise to promote cell fate transitions, Science 373 (6557) (2021) 10.1126/science.abc6506. PMC8667278 34301855

[5] Yang D, Dann C, Valdespino A, Robinson-Mailman M. Henn L., Chen M, Rosner MR Balázsi G, Analysis of gene expression heterogeneity reveals therapeutic targets and novel regulators of metastasis, 2022, 10.1101/2022.12.16.520816, bioRxiv (preprint).

[6] Kwan EK, Flowers J, Ming X, Dynamic equilibrium of cellular plasticity: The origin of diseases, Front. Ecol. Evol 11 (2023) 10.3389/fevo.2023.1077902.

[7] Becskei A, Serrano L, Engineering stability in gene networks by autoregulation, Nature 405 (6786) (2000) 590–593, 10.1038/35014651. 10850721

[8] Yang J, Antin P, Berx G, Blanpain C, Brabletz T, Bronner M, Campbell K, Cano A, Casanova J, Christofori G, Dedhar S, Derynck R, Ford HL, Fuxe J, García de Herreros G.J. Goodall A., Hadjantonakis A-K, Huang RYJ, Kalcheim C, Kalluri R, Kang Y, Khew-Goodall H. Levine Y., Liu J, Longmore GD, Mani SA, Massagué R. Mayor J., McClay D, Mostov KE, Newgreen DF, Nieto MA, Puisieux A, Runyan R, Savagner P, Stanger B, Stemmler MP, Takahashi Y, Takeichi M, Theveneau E, Thiery JP, Thompson EW, Weinberg RA, Williams ED, Xing J, Zhou BP, Sheng G, Guidelines and definitions for research on epithelial–mesenchymal transition, Nature Rev. Mol. Cell Biol 21 (6) (2020) 341–352, 10.1038/s41580-020-0237-9. 32300252 PMC7250738

[9] Filo M, Chang C-H, Khammash M, Biomolecular feedback controllers: from theory to applications, Curr. Opin. Biotechnol. 79 (2023) 102882, 10.1016/j.copbio.2022.102882. 36638743

[10] Savageau MA, Comparison of classical and autogenous systems of regulations in inducible operons, Nature 252 (5484) (1974) 546–549, 10.1038/252546a0. 4431516

[11] Rosenfeld N, Elowitz MB, Alon U, Negative autoregulation speeds the response times of transcription networks, J. Mol. Biol. 323 (5) (2002) 785–793, 10.1016/s0022-2836(02)00994-4. 12417193

[12] Camas FM, Blazquez J, Poyatos JF, Autogenous and non-autogenous control of response in a genetic network, Proc. Natl. Acad. Sci. USA 103 (34) (2006) 12718–12723, 10.1073/pnas.0602119103. 16908855 PMC1568915

[13] Nevozhay D, Adams RM, Murphy KF, Josic K, Balázsi G, Negative autoregulation linearizes the dose–response and suppresses the heterogeneity of gene expression, Proc. Natl. Acad. Sci. USA 106 (13) (2009) 5123–5128, 10.1073/pnas.0809901106. 19279212 PMC2654390

[14] Ramos AF, Hornos JEM, Reinitz J, Gene regulation and noise reduction by coupling of stochastic processes, Phys. Rev. E 91 (2015) 020701, 10.1103/physreve.91.020701. PMC4476401 25768447

[15] Cai W, Zhou W, Han Z, Lei J, Zhuang J, Zhu P, Wu X, Yuan W, Master regulator genes and their impact on major diseases, PeerJ 8 (2020) 10.7717/peerj.9952. PMC7546222 33083114

[16] Kotas M, Medzhitov R, Homeostasis, inflammation, and disease susceptibility, Cell 160 (5) (2015) 816–827, 10.1016/j.cell.2015.02.010. 25723161 PMC4369762

[17] Thakore PI, Black JB, Hilton IB, Gersbach CA, Editing the epigenome: technologies for programmable transcription and epigenetic modulation, Nature Methods 13 (2) (2016) 127–137, 10.1038/nmeth.3733. 26820547 PMC4922638

[18] Bulaklak K, Gersbach CA, The once and future gene therapy, Nat. Commun. 11 (1) (2020) 10.1038/s41467-020-19505-2. PMC7670458 33199717

[19] Jarrett AM, Faghihi D, Hormuth DA, Lima EABF, Virostko J, Biros G, Patt D, Yankeelov TE, Optimal control theory for personalized therapeutic regimens in oncology: Background, history, challenges, and opportunities, J. Clin. Med. 9 (5) (2020) 1314, 10.3390/jcm9051314. 32370195 PMC7290915

[20] Giovanini G, Barros LRC, Gama LR, Tortelli TC, Ramos AF, A stochastic binary model for the regulation of gene expression to investigate responses to gene therapy, Cancers 14 (3) (2022) 633, 10.3390/cancers14030633. 35158901 PMC8833822

[21] Peccoud J, Ycart B, Markovian modelling of gene product synthesis, Theor. Popul. Biol. 48 (2) (1995) 222–234, 10.1006/tpbi.1995.1027.

[22] Suter DM, Molina N, Gatfield D, Schneider K, Schibler U, Naef F, Mammalian genes are transcribed with widely different bursting kinetics, Science 332 (6028) (2011) 472–474, 10.1126/science.1198817. 21415320

[23] Larsson AJM, Johnsson P, Hartmanis L Hagemann-Jensen M, Faridani OR, Reinius B, Segerstolpe Å, Rivera CM, Ren B, Sandberg R, Genomic encoding of transcriptional burst kinetics, Nature 565 (7738) (2019) 251–254, 10.1038/s41586-018-0836-1. 30602787 PMC7610481

[24] Hammar P, Walldén D. Fange M., Persson F, Baltekin O, Ullman G, Leroy P, Elf J, Direct measurement of transcription factor dissociation excludes a simple operator occupancy model for gene regulation, Nature Genet. 46 (4) (2014) 405–408, 10.1038/ng.2905. 24562187 PMC6193529

[25] Du M, Kodner S, Bai L, Enhancement of laci binding in vivo, Nucleic Acids Res. 47 (18) (2019) 9609–9618, 10.1093/nar/gkz698. 31396617 PMC6765135

[26] Lammers NC, Galstyan V, Reimer A, Medin SA, Wiggins CH, Garcia HG, Multimodal transcriptional control of pattern formation in embryonic development, Proc. Natl. Acad. Sci. USA 117 (2) (2020) 836–847, 10.1073/pnas.1912500117. 31882445 PMC6969519

[27] Barkley MD, Riggs AD, Jobe A, Bourgeois S, Interaction of effecting ligands with lac repressor and repressor-operator complex, Biochemistry 14 (8) (1975) 1700–1712, 10.1021/bi00679a024. 235964

[28] Masuda LHP, Sabino AU, Reinitz J, Ramos AF, Andrioli LP MachadoLima A, Global repression by tailless during segmentation, Dev. Biol. 505 (2024) 11–23, 10.1016/j.ydbio.2023.09.014. 37879494 PMC10949167

[29] Casabon I, Zhu S, Otani H, Liu J, Mohn WW, Eltis LD, Regulation of the kstr2 regulon of mycobacterium tuberculosis by a cholesterol catabolite, Mol. Microbiol. 89 (6) (2013) 1201–1212, 10.1111/mmi.12340. 23879670

[30] Moroishi T, Park HW, Qin B, Chen Q, Meng Z, Plouffe SW, Taniguchi K, Yu F-X, Karin M, Pan D, Guan K-L, A yap/taz-induced feedback mechanism regulates hippo pathway homeostasis, Genes & Dev. 29 (12) (2015) 1271–1284, 10.1101/gad.262816.115. 26109050 PMC4495398

[31] Wilburn KM, Fieweger RA, VanderVen BC, Cholesterol and fatty acids grease the wheels of mycobacterium tuberculosis pathogenesis, Pathog. Dis 76 (2) (2018) 10.1093/femspd/fty021. PMC6251666 29718271

[32] Cao Z, Grima R, Analytical distributions for detailed models of stochastic gene expression in eukaryotic cells, Proc. Natl. Acad. Sci. 117 (9) (2020) 4682–4692, 10.1073/pnas.1910888117. 32071224 PMC7060679

[33] Prata GN, Hornos JEM, Ramos AF, Stochastic model for gene transcription on drosophila melanogaster embryos, Phys. Rev. E 93 (2) (2016) 10.1103/physreve.93.022403. 26986358

[34] Gillespie DT, A general method for numerically simulating the stochastic time evolution of coupled chemical reactions, J. Comput. Phys. 22 (1976) 403–434.

[35] Yeung K, Seitz T, Li S, Janosch P, McFerran B, Kaiser C, Fee F, Katsanakis KD, Rose DW, Mischak H, Sedivy JM, Kolch W, Suppression of raf-1 kinase activity and MAP kinase signalling by RKIP, Nature 401 (6749) (1999) 173–177, 10.1038/43686. 10490027

[36] Yun J Dangi-Garimella S, Eves EM, Newman M, Erkeland SJ, Hammond SM, Minn AJ, Rosner MR, Raf kinase inhibitory protein suppresses a metastasis signalling cascade involving LIN28 and let-7, EMBO J. 28 (4) (2009) 347–358, 10.1038/emboj.2008.294. 19153603 PMC2646152

[37] Yesilkanal AE, Yang D, Valdespino A, Tiwari P, Sabino AU, Nguyen LC, Lee J, Xie X-H, Sun S, Dann C, Steinberg E Robinson-Mailman L, Stuhlmiller T, Frankenberger C, Goldsmith E, Johnson GL, Ramos AF, Rosner MR, Limited inhibition of multiple nodes in a driver network blocks metastasis, ELife 10 (2021) 10.7554/elife.59696. PMC8128439 33973518

[38] Bonavida B, Baritaki S (Eds.), Prognostic and Therapeutic Applications of RKIP in Cancer, Academic Press, 2020, 10.1016/c2019-0-00062-3.

[39] Zhao J, Wenzel S, Interactions of RKIP with inflammatory signaling pathways, Crit. Rev. Oncog 19 (6) (2014) 497–504, 10.1615/critrevoncog. 2014011950. 25597359 PMC4299596

[40] Yeung KC, Rose DW, Dhillon AS, Yaros D, Gustafsson M, Chatterjee D, McFerran B, Wyche J, Kolch W, Sedivy JM, Raf kinase inhibitor protein interacts with NF-*κ*b-inducing kinase and TAK1 and inhibits NF-*κ*b activation, Mol. Cell. Biol. 21 (21) (2001) 7207–7217, 10.1128/mcb.21.21.7207-7217.2001. 11585904 PMC99896

[41] Datar I, Tegegne H, Qin K, Bitar MS Al-Mulla F, Trumbly RJ, Yeung KC, Genetic and epigenetic control of RKIP transcription, Crit. Rev. Oncog 19 (6) (2014) 417–430, 10.1615/critrevoncog.2014012025. 25597352

[42] Yesilkanal AE, Johnson GL, Ramos AF, Rosner MR, New strategies for targeting kinase networks in cancer, J. Biol. Chem. 297 (4) (2021) 101128, 10.1016/j.jbc.2021.101128. 34461089 PMC8449055

[43] Li D-X, Cai H-Y, Wang X, Feng Y-L, Cai S-W, Promoter methylation of raf kinase inhibitory protein: A significant prognostic indicator for patients with gastric adenocarcinoma, Exp. Ther. Med 8 (3) (2014) 844–850, 10.3892/etm.2014.1833. 25120612 PMC4113522

[44] Wei H, Liu Z, She H, Liu B, Gu J, Wei D, Zhang X, Wang J, Qi S, Ping F, Promoter methylation and expression of raf kinase inhibitory protein in esophageal squamous cell carcinoma, Oncol. Lett. 13 (3) (2017) 1866–1872, 10.3892/ol.2017.5617. 28454336 PMC5403527

[45] Beach S, Tang H, Park S, Dhillon AS, Keller ET, Kolch W, Yeung KC, Snail is a repressor of RKIP transcription in metastatic prostate cancer cells, Oncogene 27 (15) (2007) 2243–2248, 10.1038/sj.onc.1210860. 17952120 PMC2933472

[46] Labbozzetta M, Poma P, Vivona N, Gulino A, Notarbartolo M D'Alessandro N, Epigenetic changes and nuclear factor-*κ*b activation, but not microRNA-224, downregulate raf-1 kinase inhibitor protein in triple-negative breast cancer SUM 159 cells, Oncol. Lett. 10 (6) (2015) 3807–3815, 10.3892/ol.2015.3787. 26788213 PMC4665334

[47] Ren G, Baritaki S, Marathe H, Feng J, Park S, Beach S, Bazeley PS, Beshir AB, Fenteany G, Mehra R, Daignault S, Keller E Al-Mulla F, Bonavida B, de la Serna I, Yeung KC, Polycomb protein EZH2 regulates tumor invasion via the transcriptional repression of the metastasis suppressor RKIP in breast and prostate cancer, Cancer Res. 72 (12) (2012) 3091–3104, 10.1158/0008-5472.can-11-3546. 22505648

[48] Lee J, Lee J, Farquhar KS, Yun J, Frankenberger CA, Bevilacqua E, Yeung K, Kim E-J, Rosner MR Balázsi G, Network of mutually repressive metastasis regulators can promote cell heterogeneity and metastatic transitions, Proc. Natl. Acad. Sci. USA 111 (3) (2014) E364–E373, 10.1073/pnas.1304840111. 24395801 PMC3903259

[49] Baritaki S, Sahakyan A Huerta-Yepez S, Karagiannides I, Bakirtzi K, Jazirehi A, Bonavida B, Mechanisms of nitric oxide-mediated inhibition of EMT in cancer, Cell Cycle 9 (24) (2010) 4931–4940, 10.4161/cc.9.24.14229. 21150329 PMC3233484

[50] Yun J, Frankenberger CA, Kuo W-L, Boelens MC, Eves EM, Cheng N, Liang H, Li W-H, Ishwaran H, Minn AJ, Rosner MR, Signalling pathway for RKIP and let-7 regulates and predicts metastatic breast cancer, EMBO J. 30 (21) (2011) 4500–4514, 10.1038/emboj.2011.312. 21873975 PMC3230370

[51] Hayot F Iyer-Biswas S, Jayaprakash C, Stochasticity of gene products from transcriptional pulsing, Phys. Rev. E 79 (3) (2009) 10.1103/physreve.79.031911. 19391975

[52] Innocentini GCP, Hornos JEM, Modeling stochastic gene expression under repression, J. Math. Biol. 55 (3) (2007) 413–431, 10.1007/s00285-007-0090-x. 17516070

[53] Shahrezaei V, Swain PS, Analytical distributions for stochastic gene expression, Proc. Natl. Acad. Sci. USA 105 (45) (2008) 17256–17261, 10.1073/pnas.0803850105. 18988743 PMC2582303

[54] Ramos AF, Innocentini GCP, Forger FM, Hornos JEM, Symmetry in biology: from genetic code to stochastic gene regulation, IET Syst. Biology 4 (5) (2010) 311–329, 10.1049/iet-syb.2010.0058. 20831344

[55] Gama LR, Giovanini G, Ramos AF Balázsi G, Binary expression enhances reliability of messaging in gene networks, Entropy 22 (4) (2020) 479, 10.3390/e22040479. 33286254 PMC7516962

[56] Zoller B, Little SC, Gregor T, Diverse spatial expression patterns emerge from unified kinetics of transcriptional bursting, Cell 175 (3) (2018) 835–847, 10.1016/j.cell.2018.09.056. 30340044 PMC6779125

[57] Yvinec R da Silva LGS, Prata G, Dhar V, Reinitz J, Ramos AF, Two-state stochastic model of in vivo observations of transcriptional bursts, Braz. J. Phys. 55 (150) (2025) 10.1007/s13538-025-01785-y. PMC12077311 40370693

[58] Dar RD, Razooky BS, Singh A, Trimeloni TV, Cox CD McCollum JM, Simpson ML, Weinberger LS, Transcriptional burst frequency and burst size are equally modulated across the human genome, Proc. Natl. Acad. Sci. 109 (43) (2012) 17454–17459, 10.1073/pnas.1213530109. 23064634 PMC3491463

[59] Raj A, van Oudenaarden A, Nature, nurture or chance: Stochastic gene expression and its consequences, Cell 135 (2) (2008) 216–226, 10.1016/j.cell.2008.09.050. 18957198 PMC3118044

[60] Sin C McShane E, Zauber H, Wells JN, Donnelly N, Wang X, Hou J, Chen W, Storchova Z, Marsh JA, Valleriani A, Selbach M, Kinetic analysis of protein stability reveals age-dependent degradation, Cell 167 (3) (2016) 803–815, 10.1016/j.cell.2016.09.015. 27720452

[61] Kamar RI, Banigan EJ, Erbas A, Giuntoli RD, Olvera de la Cruz M, Johnson RC, Marko JF, Facilitated dissociation of transcription factors from single dna binding sites, Proc. Natl. Acad. Sci. 114 (16) (2017) 10.1073/pnas.1701884114. PMC5402408 28364020

[62] Mazzocca M, Colombo E, Callegari A, Mazza D, Transcription factor binding kinetics and transcriptional bursting: What do we really know? Curr. Opin. Struct. Biol. 71 (2021) 239–248, 10.1016/j.sbi.2021.08.002. 34481381

[63] Bilgin B, Nath A, Chan C, Walton SP, Characterization of transcription factor response kinetics in parallel, BMC Biotechnol. 16 (1) (2016) 10.1186/s12896-016-0293-6. PMC4997724 27557669

[64] Foreman R, Wollman R, Mammalian gene expression variability is explained by underlying cell state, Mol. Syst. Biology 16 (2) (2020) 10.15252/msb.20199146. PMC7011657 32043799

[65] Keren L, Hausser J, Alisar H Lotan-Pompan M, Kaminski S, Weinberger A, Alon U, Milo R, Segal E, Massively parallel interrogation of the effects of gene expression levels on fitness, Cell 166 (5) (2016) 1282–1294, 10.1016/j.cell.2016.07.024. 27545349

[66] Yesilkanal A, Rosner M, Targeting raf kinase inhibitory protein regulation and function, Cancers 10 (9) (2018) 306, 10.3390/cancers10090306. 30181452 PMC6162369

[67] Gillespie DT, Exact stochastic simulation of coupled chemical reactions, J. Phys. Chem. 81 (1977) 2340–2361.

[68] Dyson FJ, The radiation theories of tomonaga, schwinger, and feynman, Phys. Rev. 75 (3) (1949) 486–502, 10.1103/physrev.75.486.

[69] Rugh WJ, Linear System Theory, second ed., in: Prentice Hall information and system sciences series, Prentice Hall, Upper Saddle River, NJ, 1996.

[70] Giovanini G, Sabino AU, Barros LRC, Ramos AF, A comparative analysis of noise properties of stochastic binary models for a self-repressing and for an externally regulating gene, Math. Biosci. Eng. 17 (5) (2020) 5477–5503, 10.3934/mbe.2020295. 33120562

[71] Purvis JE, Lahav G, Encoding and decoding cellular information through signaling dynamics, Cell 152 (5) (2013) 945–956, 10.1016/j.cell.2013.02.005. 23452846 PMC3707615

[72] Levine JH, Lin Y, Elowitz MB, Functional roles of pulsing in genetic circuits, Science 342 (6163) (2013) 1193–1200, 10.1126/science.1239999. 24311681 PMC4100686

[73] Kirkpatrick S, Gelatt CD, Vecchi MP, Optimization by simulated annealing, Science 220 (4598) (1983) 671–680, 10.1126/science.220.4598.671. 17813860

[74] Chu K-W, Deng Y, Reinitz J, Parallel simulated annealing by mixing of states, J. Comput. Phys. 148 (2) (1999) 646–662, 10.1006/jcph.1998.6134.

[75] Chubb JR, Trcek T, Shenoy SM, Singer RH, Transcriptional pulsing of a developmental gene, Curr. Biol. 16 (10) (2006) 1018–1025, 10.1016/j.cub.2006.03.092. 16713960 PMC4764056

[76] Trudeau RT Leyes Porello EA, Lim B, Transcriptional bursting: stochasticity in deterministic development, Development 150 (12) (2023) 10.1242/dev.201546. PMC10323239 37337971

[77] Fukaya T, Enhancer dynamics: Unraveling the mechanism of transcriptional bursting, Sci. Adv. 9 (31) (2023) 10.1126/sciadv.adj3366. PMC10396287 37531441

[78] Kandavalli V, Zikrin S, Elf J, Jones D, Anti-correlation of laci association and dissociation rates observed in living cells, Nat. Commun. 16 (1) (2025) 10.1038/s41467-025-56053-z. PMC11748676 39824877

[79] Carthew RW, Sontheimer EJ, Origins and mechanisms of mirnas and sirnas, Cell 136 (4) (2009) 642–655, 10.1016/j.cell.2009.01.035. 19239886 PMC2675692

[80] Zhu Y, Zhu L, Wang X, Jin H, Rna-based therapeutics: an overview and prospectus, Cell Death & Dis. 13 (7) (2022) 10.1038/s41419-022-05075-2. PMC9308039 35871216

[81] Ali Kulsoom W, Wang F, Advancement in synthetic gene circuits engineering: An alternative strategy for microrna imaging and disease theranostics, Biotech. Adv 79 (2025) 108518, 10.1016/j.biotechadv.2025.108518. 39798857

[82] Wan Y, Cohen J, Szenk M, Farquhar KS, Coraci D, Azukas J Krzysztoń R, Smashnov A Van Nest N, Chern Y-J, Nguyen LC De Martino D, Bien H, Chan C-H Bravo-Cordero JJ, Rosner MR, Balázsi G, Nonmonotone invasion landscape by noise-aware control of metastasis activator levels, Nat. Chem. Biology 19 (7) (2023) 887–899, 10.1038/s41589-023-01344-z. PMC10299915 37231268

[83] Stortz M, Presman DM, Levi V, Transcriptional condensates: a blessing or a curse for gene regulation? Commun. Biology 7 (1) (2024) 10.1038/s42003-024-05892-5. PMC10873363 38365945

[84] Dejosez M, Ramamoorthy M Dall'Agnese A, Platt J, Yin X, Hogan M, Brosh R, Weintraub AS, Hnisz D, Abraham BJ, Young RA, Zwaka TP, Regulatory architecture of housekeeping genes is driven by promoter assemblies, Cell Rep. 42 (5) (2023) 112505, 10.1016/j.celrep.2023.112505. 37182209 PMC10329844

[85] Fujita J, Freire P, Coarfa C, Benham AL, Gunaratne P, Schneider MD, Dejosez M, Zwaka TP, Ronin governs early heart development by controlling core gene expression programs, Cell Rep. 21 (6) (2017) 1562–1573, 10.1016/j.celrep.2017.10.036. 29117561 PMC5695914

[86] Wei Y, Zhang Y, Cao W, Cheng N, Xiao Y, Zhu Y, Xu Y, Zhang L, Guo L, Song J, Sha S, Shao B, Ma F, Yang J, Ying Z, He Z, Chai R, Fang Q, Yang J, Ronin/hcf1-tfeb axis protects against d-galactose-induced cochlear hair cell senescence through autophagy activation, Adv. Sci 12 (29) (2025) 10.1002/advs.202407880. PMC12362728 39985193

[87] Thieffry D, Huerta AM, Collado-Vides J Pérez-Rueda E, From specific gene regulation to genomic networks: a global analysis of transcriptional regulation in escherichia coli, BioEssays 20 (5) (1998) 433–440, 10.1002/(sici)1521-1878(199805)20:5<433::aid-bies10>3.0.co;2-2. 9670816

[88] Milo R Shen-Orr SS, Mangan S, Alon U, Network motifs in the transcriptional regulation network of escherichia coli, Nat Genet. 31 (1) (2002) 64–68, 10.1038/ng881. 11967538

[89] Senn G, Nissen L, Benenson Y, Synthetic gene circuits that selectively target ras-driven cancers, ELife (2025) 10.7554/elife.104320.2. PMC12931925 41733988

[90] Rullan M Milias-Argeitis A, Aoki SK, Buchmann P, Khammash M, Automated optogenetic feedback control for precise and robust regulation of gene expression and cell growth, Nat. Commun. 7 (1) (2016) 10.1038/ncomms12546 PMC5007438 27562138

