# Supplementary material for: A control theoretical approach to gene regulation reveals quantitative constraints for dynamic homeostasis in stochastic gene expression
Source: Biochim Biophys Acta Mol Basis Dis. Author manuscript; Available in PMC 2026 Jun 20. (PMC13282713; doi:10.1016/j.bbadis.2026.168219)
Supplement: 1 [file NIHMS2180027-supplement-1.pdf]

Supporting Information for "A control theoretical approach to gene regulation reveals quantitative constraints for dynamic homeostasis in stochastic gene expression"

Guilherme Giovanini[a], Cyro von Zuben de Valega Negrão[b], Ammar Alsinai[c], Marsha Rich Rosner[d], Gábor Balázsi[e,f] Alexandre Ferreira Ramos[g,h,1]

[a]Departamento de Radiologia e Oncologia , Instituto do Câncer do Estado de São Paulo (ICESP), Hospital das Clínicas da Faculdade de Medicina da Universidade de São Paulo (HCFMUSP), São Paulo, 01246-000, SP, Brazil [b]Brazilian Biosciences National Laboratory (LNBio), Brazilian Center for Research in Energy & Materials (CNPEM), Campinas, 13083-970, SP, Brazil [c]Department of Computer Science, College of Engineering and Information Technology, Onaizah Colleges, Unaizah, 56447, Qassim, Saudi Arabia [d]Ben May Department for Cancer Research, The University of Chicago, Chicago, 60637, IL, United States [e]The Louis and Beatrice Laufer Center for Physical and Quantitative Biology, Stony Brook University, Stony Brook, 11794, NY, United States [f]Department of Biomedical Engineering, Stony Brook University, Stony Brook Cancer Center, Stony Brook 11794, NY, United States [g]Comprehensive Center for Precision Oncology, Instituto do Câncer do Estado de São Paulo (ICESP), Hospital das Clínicas da Faculdade de Medicina da Universidade de São Paulo (HCFMUSP), São Paulo, 01246-000, SP, Brazil [h]Escola de Artes, Ciências e Humanidades, Universidade de São Paulo, São Paulo, 03828-000, SP, Brazil; [1]To whom correspondence should be addressed. E-mail: alex.ramos@usp.br

Supporting Information Figures

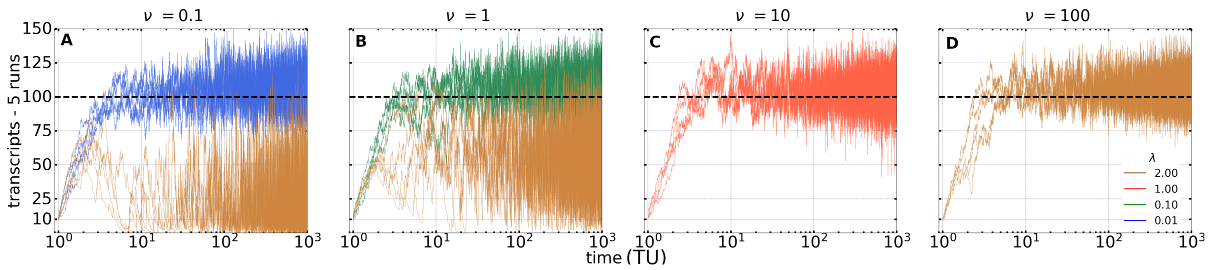

Fig. S1. SSA trajectories of RNA levels regulated at homeostasis by sampling rate ν. Graphs display RNA levels trajectories, which the columns present increasing sampling rate ν, from left to right. All curves follow the color key within **(D)** for four different decaying rates λ of feedback surges. Five SSA curves are shown for each λ within graphs **(A–D)**. A dashed line at 100 represents the aimed average RNA number $\overline{M}$. The scales of the rates and time are relative to the mRNA degradation rate ρ.

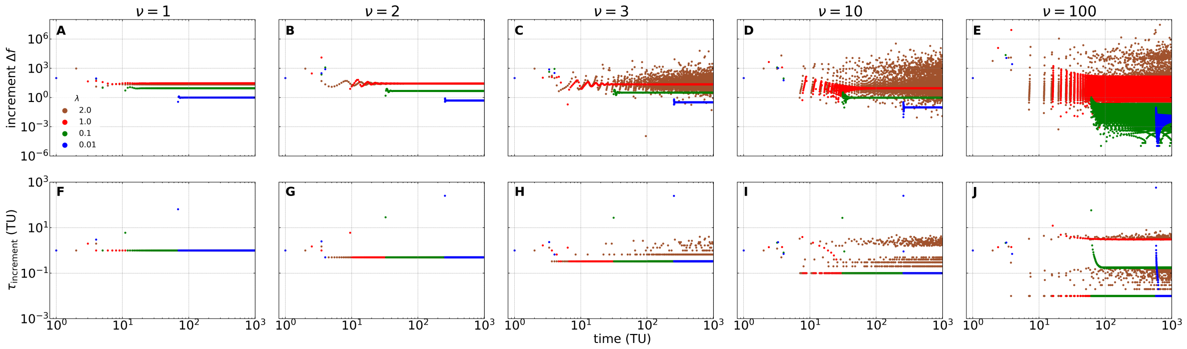

Fig. S2. Control of mRNA levels at large sampling rates depends on a non-monotonic agenda of increments. The upper row **(A–E)** and lower row **(F–J)**, respectively, depict trajectories of the intensities and time intervals of the feedback surges. From left to right, the trajectories correspond to the increasing sampling rate ν. Note that the scales of the rates and time are relative to the mRNA degradation rate ρ, and all axes are in the logarithm scale.
